# Epigenetic modifications as key regulators of Waldenstrom's Macroglobulinemia biology

**DOI:** 10.1186/1756-8722-3-38

**Published:** 2010-10-07

**Authors:** Antonio Sacco, Ghayas C Issa, Yong Zhang, Yang Liu, Patricia Maiso, Irene M Ghobrial, Aldo M Roccaro

**Affiliations:** 1 Dana-Farber Cancer Institute, Medical Oncology, Harvard Medical School, Boston, MA, USA

## Abstract

Waldenstrom's Macroglobulinemia is a low-grade B-cell lymphoma characterized by the presence of lymphoplasmacytic cells in the bone marrow and a monoclonal immunoglobulin M in the circulation. Recent evidences support the hypothesis that epigenetic modifications lead to Waldesntrom cell proliferation and therefore play a crucial role in the pathogenesis of this disease. Indeed, while cytogenetic and gene expression analysis have demonstrated minimal changes; microRNA aberrations and modification in the histone acetylation status of primary Waldenstrom Macroglobulinemia tumor cells have been described. These findings provide a better understanding of the underlying molecular changes that lead to the initiation and progression of this disease.

## Introduction

First introduced by C.H. Waddington in 1939 to name "the causal interactions between genes and their products, which bring the phenotype into being", the term "epigenetics" was later defined as heritable changes in gene expression that are not due to any alteration in the DNA sequence [1]. The best-known epigenetic markers are DNA methylation and histone acetylation. Moreover, all these phenomena are finely regulated in different manners, such as through microRNAs (miRNAs) [2].

Waldenström's Macroglobulinemia (WM) is a low-grade B-cell lymphoma characterized by the presence of lymphoplasmacytic cells in the bone marrow (BM) and a monoclonal immunoglobulin M in the circulation [3,4]. The most common risk factor for WM is IgM MGUS (monoclonal gammopathy of undermined significance), which is associated with the presence of a small IgM protein in the circulation and minimal involvement (< 10% lymphoplasmacytic cells) in the bone marrow, with the absence of symptoms or signs of the disease. The risk of progression of IgM MGUS is the highest in all types of MGUS with about 5-10% risk of progression per year.

**Figure 1:**
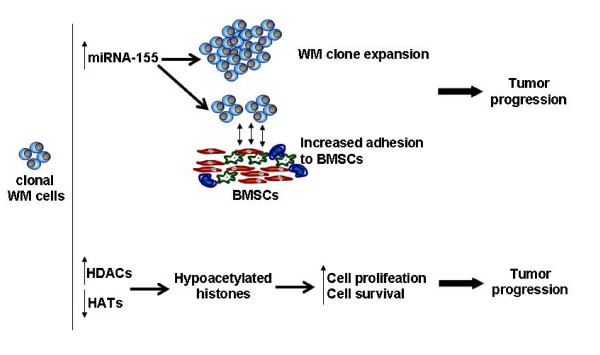
**miRNA aberrations and hypoacetylated histones drive WM pathogenesis**. WM tumor cells present with increased level of miRNA-155, together with increased expression of HDACs and reduced expression of HATs, leading WM clone expansion and tumor progression. (BMSCs: bone marrow stromal cells: miRNA-155: microRNA-155; HDAC: histone deacetylase; HAT: histone acetyltransferase)

While WM cells showed minimal changes at cytogenetic studies and gene expression analysis [5], primary WM tumor cells present with a miRNA signature that differentiates them from their normal counterpart. Among deregulated miRNAs, miRNA-155 has been shown to play a pivotal role in the biology of this disease both in vitro and in vivo. Moreover, other miRNA changes in WM cells, such as the down-regulation of miRNA-9*, has been proven to modulate the histone acetylation status in WM cells [6]. These findings support the idea that epigenetic modifications are crucial in the pathogenesis of WM. Importantly, these observation provide the preclinical rational for testing miRNA-based therapeutical approaches for the treatment of WM disease (Figure 1).

In this review, we will focus on the role of miRNAs and histone acetylation as key regulators of WM biology.

### miRNA signature characterizes primary WM tumor cells

miRNAs constitute a class of small, non-coding, 18-24 nucleotide RNAs, described for the first time in the nematode *Caenorhabditis elegans *[7]. By repressing several target mRNAs, mature miRNAs play a pivotal role in regulating development, cell differentiation, apoptosis, and cell proliferation [8-10]. Importantly, miRNAs have been described to play roles in both solid tumors and hematologic malignancies [11-13]. We have recently performed miRNA studies in WM, and through unsupervised clustering, we showed that WM clonal cells present with a miRNA signature that differs from that of their normal counterpart. Importantly, seven miRNAs were specifically different between WM patients and healthy individuals: among them, miRNA-363*, -206, -494, -155, -184, -542-3p demonstrated increased expression in WM patients; whereas miRNA-9* demonstrated decreased expression in WM patients. Predicted targets of the increased miRNAs in WM patients included tumor suppressors, cell cycle inhibitors, cytokine signaling suppressors and tyrosine phosphatases. Conversely, predicted target genes for the decreased miRNAs in WM included protein kinases, oncogenes and transcription factors. In addition, we showed that several protein kinases, transcription factors, and cell cycle regulators were up-regulated in WM patients including protein kinase C, serine/threonine kinase 24, mitogen-activated protein kinase kinase kinase 7, E2F- and pre-B-cell leukemia-transcription factors, as well as cyclin D2 and cyclin-dependent kinase inhibitor [14], suggesting that miRNAs may play a crucial role in WM pathogenesis.

miRNA-155 is expressed from an exon of the non-coding BIC gene [15]. Its role as oncogenic miRNA and its involvement in the initiation and progression of cancers has been reported in several hematologic malignancies, including diffuse large B-cell lymphomas, chronic lymphocytic leukemia and primary mediastinal B-cell lymphomas, where it has shown to be highly expressed [16-18]. Based on the evidence that miRNA-155 is increased in primary WM cells, we subsequently conducted in vitro and in vivo studies to better define the functional and biological role of miRNA-155 in supporting WM cell pathogenesis; and it has been demonstrated that DNA synthesis was significantly reduced in miRNA-155 knocked-down cells compared to control cells [14]. Similarly, miRNA-155 modulated cell cycle progression in WM cells, as demonstrated by an increased % of cells in G1-phase, together with a decreased % of cells in S-phase. These findings were validated at gene expression profiling that showed increased expression of cyclin-dependent kinase inhibitors (p18, p19, p21, p27); decreased expression of cyclin-dependent kinases-2, -4, -6, as well as cyclins D1, D2, D3 and E; and increased expression of p53 with decreased expression of its negative regulator (Mdm2) in the miRNA-155 knockdown WM cells compared to controls. In addition, the impact of miRNA-155 knockdown on signaling cascades regulating proliferation was next investigated showing that miRNA-155 knockdown strongly inhibited ERK- and AKT-phosphorylation, as well as p-GSK3α/β and p-S6R, both AKT downstream target proteins.

It has been clearly demonstrated that BM microenvironment confers growth advantages to and induces drug resistance in malignant cells [19]. Therefore, the role of miRNA-155 in regulating WM cell proliferation in the context of BM milieu has been investigated, showing a significant inhibition of adhesion to fibronectin in miRNA-155 knockdown cells compared to controls. These findings were also supported by downregulation of genes such as Rho GTPase activating proteins, p21(CDKN1A) activating protein, and p21-activated kinase (PAK)-1 interacting protein, which are known to be involved in the adhesion process [20]. Similarly, miRNA-155 knockdown significantly inhibited WM cell migration in response to stromal derived factor-1 (SDF-1), an important regulator of migration in B cells. In addition, while adherence of both non-transfected or control probe-transfected WM cells to BM stromal cells triggered 34% and 33% increase in proliferation, any significant increase in the proliferation rate of miRNA-155 knocked down WM cells has been described, indicating that they overcome the growth advantage from of BM milieu. To better support the in vitro findings, effect of miRNA-155 in regulating WM cell migration was also confirmed in vivo, by using an in vivo homing model [21]. In addition, mice injected with miRNA-155 knockdown probe-transfected WM cells had prolonged survival compared with mice injected with control probe-transfected WM cells, with mean survival of 20 days vs 14 days (P < .001) and overall survival of 21 days *vs *15 days, respectively.

### WM cells present with a hypoacetylated status of histone, partially driven by miRNA aberrations in the clonal WM cell population

Histone acetylation is commonly deregulated in several tumors. Nucleosomal histone acetylation homeostasis is responsible for the transcriptional regulation of many genes: particularly, hypoacetylation is associated with a condensed chromatin structure leading to the repression of gene transcription; while, hyperacetylation is associated with a more open chromatin structure and activation of transcription [22,23]. This balance is modulated by a tight regulation of histone deacetylase (HDAC) and histone acetyl transferases (HATs) levels. In many malignancies, this balance is deregulated; and tumor cells present with an increased expression of HDACs leading to decreased gene transcription [7,8]. HDACs are enzymes that catalyze the removal of the acetyl modification on lysine residues of proteins, including the nucleosomal histones H2A, H2B, H3, and H4. In addition, other induced genes include the cell cycle kinase inhibitor p21^WAF1^, p16^ink4a^, and p27^Kip^, p53, NF-YA, and GATA-1, leading to enhanced cellular functions such as proliferation, cell-cycle, and survival [24].

Alterations in the balance between HAT and HDAC activity in many cancers will lead to deregulated gene expression and the induction of proliferation and survival in tumor cells [25,26]. HDACs mediate the function of oncogenic translocations in many malignancies including promyelocytic (PML)-retinoic acid receptor alpha (RARα) in acute promyelocytic leukemia [27]. Most of the aberrant HAT and HDAC activity has been due to translocation, amplification, overexpression or mutation in many malignancies, including hematological malignancies [25,26,28]. Recent studies have demonstrated that miRNAs may exert their activity by interfering with the epigenetic machinery, such as modulating the expression of enzymes regulating DNA methylation or histone modification [27-30]. For example, it has been demonstrated that up-regulation of miRNA-449a in prostate cancer cells exerts an anti-proliferative effect on the tumor clone, supported by cell cycle arrest and induction of a senescence-like phenotype and apoptosis [30]. In addition, other miRNAs are responsible for targeting histone methyltransferases. It has been recently reported that miRNA-101 targets the enhancer of Zeste homolog 2 (EZH2); the low expression level in several tumor types could lead to up-regulation of EZH2 in aggressive tumors with an invasive phenotype [31,32].

Recent studies have focused on the histone acetylation status in WM cells and how miRNA may be responsible for regulating histone acetylation in this disease. miRNA-206 and -9* are respectively increased and decreased in WM cells, as compared to normal cells [14]. Predicted targets for the increased miRNA-206 and decreased miRNA-9* included histone-acetyltransferases (HATs) and HDACs, respectively, suggesting a possible role of miRNA-206 and -9* in regulating histone acetylation in WM. Indeed, primary WM cells were characterized by significant increased expression of HDAC-2, -4, -5, -6, -8, -9, and significant decreased expression of HAT-1, -2, and -3; together with a significantly higher HDAC activity as compared to controls. Importantly, functional studies revealed that acetyl histone-H3 and -H4 were upregulated in pre-miRNA-9*- and -anti-miRNA-206-transfected cells, with a higher acetyl histone-H3 and -H4 up-regulation upon miRNA-9* modulation. Moreover, miRNA-9*-dependent modulation of HDAC activity lead to reduced WM cell proliferation and increased WM cell toxicity, suggesting a key role of miRNA-9* in regulation WM progression [6].

## Conclusions

Modifications at the level of miRNAs have recently gained considerable attention in the field of cancer research. It has been recently identified an increased expression of miRNAs-363*, -206, -494, -155, -184, -542-3p, and decreased expression of miRNA-9* in primary bone marrow-derived WM tumor cells. Based on this first observation, functional studies have been focused on miRNA-155, and the oncogenic role of miRNA-155 has been confirmed and validated in WM cells. Indeed, miRNA-155 acts as a critical regulator of proliferation of WM cells, where it specifically targets WM cells even in the context of a bone marrow milieu. This provides preclinical evidences to enhance our understanding about the role of epigenetic changes in WM pathogenesis. Indeed, if cytogenetic and molecular studies on gene expression analysis at the miRNA level have demonstrated minimal changes in WM cells, the described significant differences in WM miRNA expression profiling improve our understanding of the underlying molecular changes that lead to the initiation and progression of this rare disease. Importantly, miRNA-155 may be regarded as a sufficiently restricted therapeutic target in WM.

In addition, subsequent studies have demonstrated that that loss of miRNA-9* may be responsible for up-regulation of HDAC4 and HDAC5 in primary WM cells, contributing to the pathogenesis of this disease; this also indicates the potential therapeutic value of synthetic miRNA oligonucleotides as epigenetic modulators with a mechanism of action similar to chemical HDAC inhibitors.

## Authors' contributions

AS, GCI, YZ, YL, PM: wrote the manuscript.

IMG, AMR: revised the manuscript.

All authors read and approved the final manuscript.

## Competing interests

The authors declare that they have no competing interests.
